# Establishment of a WHO Reference Reagent for anti-Mullerian hormone

**DOI:** 10.1186/s12958-020-00641-9

**Published:** 2020-08-15

**Authors:** Jackie Ferguson, Jason Hockley, Peter Rigsby, Chris Burns

**Affiliations:** 1 Biotherapeutics Division, National Institute for Biological Standards and Control, South Mimms, Potters Bar, Hertfordshire, UK; 2 Biostatistics Group, National Institute for Biological Standards and Control, South Mimms, Potters Bar, Hertfordshire, UK

**Keywords:** Anti-Mullerian hormone, Assay, Immunoassay, International standard, Reference material, WHO

## Abstract

**Background:**

There is a need for a reference material to support the development and ensure the quality of immunoassays for human AMH. A batch of ampoules, coded 16/190, containing lyophilised recombinant AMH was evaluated in a WHO Collaborative Study. The aims of the study were to determine the AMH content in terms of the calibration of each immunoassay method, to predict long-term stability and to assess the suitability of the preparation to calibrate AMH immunoassays.

**Methods:**

Study participants were asked to report the AMH content of specific dilutions of coded ampoules of 16/190 and a comparator preparation containing approximately half the AMH content. In each assay, participants also reported the AMH content of 22 patient samples to assess commutability. A robust all-laboratory geometric mean of the content estimates was determined using the laboratory geometric mean estimates. Commutability was assessed using a difference in bias approach. Stability was predicted by the measurement of thermally accelerated degradation samples.

**Results:**

Seven laboratories performed twenty-one immunoassay method-platform combinations, sixteen of which provided data which met the validity criteria, giving a consensus geometric mean estimate of AMH content of 511 ng/ampoule (95% CI, 426–612, *n* = 16, GCV 42%) and a robust geometric mean of 489 ng/ampoule. By contrast, the GCV% for the all-laboratory geometric mean of the relative content estimates for the comparator sample to 16/190 was 12%. Commutability was assessed using 20 of the 22 representative patient samples. Of the valid assays, 16/190 was within the limits of acceptable commutability for 6 methods, partially commutable for a further 3 methods and non-commutable when measured by 7 methods. The preparation was predicted to be highly stable when stored at − 20 °C.

**Conclusion:**

The majority of methods met the validity criteria. Content estimates showed a high between-method variability, yet assays exhibited a similar proportionality of response as demonstrated using the comparator sample. 16/190 was commutable in some but not all methods. On the basis of these results, it was agreed by the WHO Expert Committee on Biological Standardization to establish 16/190 as a WHO Reference Reagent for AMH with a content defined by consensus immunoassay of 489 ng/ampoule.

## Background

In 2014, the WHO Expert Committee on Standardization (ECBS) approved the project to develop an International Standard for Mullerian Inhibiting Substance, also known as Anti-Mullerian Hormone (AMH) for immunoassay [1]. This was in response to repeated requests [2–4] from manufacturers and the clinical community who recognised both that there was an increase in the use of serum AMH measurement and also, that there was a discordance between the AMH measurements reported by the small number of immunoassays available at the time. As serum AMH concentration is considered a biomarker which represents the dynamic reserve of developing ovarian follicles, a major clinical use of AMH measurement is to assess female infertility and to gauge the likely response to ovarian stimulation procedures for assisted reproduction. Recently, there has been an increase in the number of manufacturers who offer, or who are in the process of developing, AMH immunoassays. This has led the WHO to approve the development of a WHO Reference Reagent for AMH (NIBSC code 16/190) with the aim of providing a reference preparation to further investigate the calibration and performance of assay methods.

Produced in ovarian granulosa cells, human AMH (NCBI RefSeq NP_000470.2) is a disulphide-linked homodimer comprising two subunits of 560 amino acids, thereby having a formula mass of 113 kDa for the dimer. Proteolytic cleavage of the homodimer forms N-terminal and C-terminal disulphide-linked dimers which remain non-covalently associated. To our knowledge, commercial immunoassays detect total AMH, either uncleaved, or cleaved but with the N- and C-terminal dimers remaining associated. Each subunit is glycosylated and it is likely that this property of AMH, specifically the effect of glycosylation in retarding migration by non-reducing SDS-PAGE, which has resulted in the accepted convention that the formula mass of AMH is 140 kDa [5]. There are two potential N-linked glycosylation motifs in the amino acid sequence of AMH [6] and Pankhurst and McLennan [7] have suggested that the native protein may also contain O-linked glycosylation. As noted by Nelson and La Marca [2], as a glycosylated protein, the molecular mass of AMH is difficult to ascertain. Despite this, a molecular mass of 140 kDa is used for the widely cited conversion of 1 ng/ml = 7.14 pmol/L AMH and immunoassay methods report in mass or concentration units. The above conversion is cited by manufacturers, used by monitoring organisations (such as UK NEQAS) and there is awareness of the units and the conversion in patient groups.

The uncertainty of the true molecular mass of AMH demonstrates a key challenge presented by the accepted convention of reporting AMH immunoassays in mass units despite the measurand being a complex biological molecule with undefined post-translational modifications. The origin of the “nanogram of AMH” is unclear and the methods by which mass values were assigned to the first CHO-derived recombinant AMH immunoassay standards have not been reported [8, 9]. The unclear traceability of the mass assignment of AMH from the original assays to current assays has been described extensively elsewhere [10, 11]. By necessity, the calibration of new assay methods has relied on the use of serum value transfer using panels of human samples to transfer the AMH measurement from an AMH immunoassay that is considered the current market leader or ‘gold standard’ to the calibrators of the new assay. It is not known whether this process, over more than 15 years of assay development, has led to a situation where the current AMH content of a stated “nanogram of AMH” is different from the original measurements that described a nanogram of recombinant AMH. In situations such as this, where there is no formally established metrological traceability, one possible solution is to define an arbitrary unit of AMH immunoreactivity such as a WHO International Unit (IU) which is defined by the content of the current International Standard (IS) as determined through a WHO collaborative study [12]. However, the maturity of AMH measurement and the historic confusion caused by the discordant values reported by the first commercially available assays has led to the use of mass units being the accepted convention. If an IU of AMH immunoreactivity was established, there is a strong possibility that assay-specific conversion factors would be introduced to enable continued reporting of mass units.

However, foregoing the establishment of an IU of AMH immunoreactivity for an alternative approach, presents challenges. Technically, the assignment of mass units to a reference material requires the use of Système International (SI)-traceable physicochemical methods which precludes the use of measurement procedures with a biological component [13]. For proteins, assignment in SI units with unambiguous traceability has been successfully achieved only for small, unmodified proteins such as growth hormone (22 kDa) [14] and insulin (5.8 kDa) [15, 16]. Furthermore, once physicochemical reference methods have been developed to accurately measure AMH, for instance tryptic peptide isotope dilution mass spectrometry [17], there is still the question of which formula mass to use to convert the molar quantities reported by these methods to mass units.

The challenges highlighted above, outline the context in which we proceeded to formulate and manufacture the reference material coded NIBSC 16/190. Building on work previously undertaken to develop and assess a stable, lyophilised formulation of AMH, [11], the batch of ampoules coded 16/190 comprised recombinant CHO-derived AMH, lyophilised from a formulation containing trehalose and casein. Ampoules of 16/190 were then evaluated in a WHO international collaborative study, the results of which are summarised here. Full details of the results of the collaborative study are reported to the WHO ECBS as part of the process to establish a WHO reference material. This report, WHO/BS/2019.2363, is available on-line [18].

## Methods

### Materials

CHO-derived, recombinant AMH purified from the culture media of stable cell line LR-MIS, was donated to NIBSC by Professor Patricia Donahoe (Director) and Dr. David Pépin of the Pediatric Surgical Research Laboratories, Massachusetts General Hospital, USA. In this cell line, the coding sequence for human AMH sequence has been modified at amino acids 423–428 from RAQR/S to RARR/S to improve cleavage at this position to produce the active form of the protein [19]. Bovine casein from herds certified as BSE free was obtained from Calbiochem (San Diego, USA). Trehalose was obtained from Sigma-Aldrich (Poole, UK).

### Preparation of the candidate reference material

The content of the donated preparations of AMH was estimated using an AMH Gen II ELISA (Beckman-Coulter, High Wycombe, UK) before adding at a nominal concentration of approximately 1 μg/ml to a 2100 ml volume of formulation buffer containing 0.24% (w/v) casein and 0.5% (w/v) trehalose. The bulk formulation was distributed into 3 mL siliconized glass ampoules at 0.5 mL per ampoule. After lyophilization and sealing under nitrogen according to procedures described by WHO [13], the batch, coded 16/190 was stored at − 20 °C.

### Preparation of a comparator sample

To assist with the evaluation of the candidate preparation, a comparator sample containing approximately half the content of AMH as the candidate, 16/190, was prepared. For this, formulation containing approximately 0.5 μg/ml AMH, 0.24% (w/v) casein and 0.5% (w/v) trehalose was filled at 0.5 mL per ampoule and lyophilised as described above. In the collaborative study, the sample was coded and participants were not informed of the reduced content in the study protocol.

### WHO collaborative study

The candidate preparation, 16/190, was assessed through a WHO collaborative study involving 7 laboratories in 4 countries (Table 1) to assign a value and to evaluate its suitability and stability. Participants were provided with ampoules of the candidate preparation, 16/190, coded A, the comparator sample, coded B, containing approximately half the AMH content and a coded duplicate of 16/190, coded C. Participants were also provided with aliquots of 17 human serum samples and 5 human plasma samples with estimated AMH concentrations of < 0.1 to > 12 ng/mL. A subset of participants was provided with thermal degradation samples in which ampoules had been incubated at 4°, 20°, 37° and 45 °C for 21 months and 13 days.

**Table 1:** Participants in the WHO Collaborative Study to evaluate the proposed reference material for AMH, 16/190

CHINA	Dr Yu Ting, National Institutes for Food and Drug Control, Institute for Medical Devices Control, No. 2 Tiantan Xili, Dongcheng District, Beijing, 100,050.
FRANCE	Dr Nathalie Ripoll, bioMerieux, Chemin de L’Orme, 69,280 Marcy L’Etoile.
GERMANY	Dr Verena Hofmann, Roche Diagnostics GmbH, Nonnenwald 2, D-82377 Penzberg.
USA	Dr Patrick Sluss, Ansh Labs, 455 Medical Centre Blvd, Webster, TX 77598
USA	Ryan Masica, Beckman Coulter Inc., 1000 Lake Hazeltine Drive, Chaska, MN 55318–1084
USA	Drs Hubert Vesper, Uliana Danilenko and Candice UlmerCentre for Disease Control, Chamblee Campus Warehouse, 3719 N Peachtree Road, Chamblee, GA 30341–2251.
USA	Dr Jelena Bogdanovic, Siemens Healthcare Diagnostics, 511 Benedict Avenue, Tarrytown, NY 10591

Participants are listed in order of country

Participants were asked to perform a minimum of two independent assays in which the AMH content of a dilution series of each coded sample was measured, in triplicate, alongside triplicate measurements of the AMH content of the serum and plasma samples. Where sample number was limited by the assay format, duplicate measurements were performed. The (nominal) test concentrations of each coded sample were defined in the study protocol such that a reported, all-laboratory geometric mean for each concentration could be calculated. Thermal degradation samples were coded D-H and were treated in the same manner as the candidate materials.

### Statistical analysis

#### Assessment of the immunoreactivity of the candidate standard, 16/190

Analyses were performed with AMH concentrations as reported by the participants, using results from samples A, B and C diluted to the nominal concentration range of 0.125–16 ng/ml only. To determine if a method showed acceptable dilutional linearity, linear regression analysis was applied to each sample in each assay run to estimate the slope of log_10_ reported concentration against log_10_ nominal concentration. The r^2^ value was confirmed to exceed 0.975 in all cases. Methods were considered invalid if the geometric mean slope for any of samples A, B or C was outside the range [0.91, 1.10]. In addition, an individual assay run was only concluded to be acceptable if the ratio of the coded duplicates [A:C] was between 0.91 and 1.10.

Results from all valid methods were corrected for dilution factor and combined to generate unweighted geometric mean (GM) estimates for each laboratory and these laboratory means were used to calculate overall unweighted geometric mean estimates. Variability between laboratories has been expressed using geometric coefficients of variation (GCV = {10^s^-1} × 100% where s is the standard deviation of the log_10_ transformed estimates). To mitigate the influence of possible outliers and anomalous results, Huber’s robust geometric mean was also calculated using the R package ‘WRS2’ [20].

#### Assessment of stability

The relative immunoreactivities of the accelerated thermal degradation samples were used to fit an Arrhenius equation relating degradation rate to absolute temperature assuming first-order decay [21], and hence predict the degradation rates when stored at a range of temperatures*.*

#### Assessment of commutability

Commutability was assessed using a difference in bias approach by which, for each laboratory, the bias between the reported values for the patient samples and the consensus all-laboratory value for these samples, was compared with the bias between the reported values for the dilutions of the candidate preparation and the all-laboratory consensus values for each dilution.

*Data used for analysis.* AMHPlasma18 and AMHPlasma22 were excluded from the analysis as the AMH content was below or above, respectively, the range of some assays (*n* = 9 for AMHPlasma18, *n* = 7 for AMHPlasma22). All reported results were log_10_ transformed for analysis in order to achieve approximately constant scatter over the range of concentrations used. A consensus value for each sample was calculated as Huber’s robust mean of laboratory means using the R package ‘WRS2’ [20]. Bias values were then calculated for all reported results as the difference between the reported value and the study consensus value for that sample.

*Determination of commutability criteria.* The standard deviation of the bias values for patient samples was calculated within each laboratory and a pooled value, *s*_*P*_, was calculated across all laboratories. Commutability criteria representing the maximum acceptable difference in bias were then set as ±2*s*_*P*_. Reference standards were to be concluded as commutable if the observed bias was within the commutability criteria. For this commutability assessment, the bias for patient samples has been assumed to be constant over the concentration range used.

## Results

### Preparation of the candidate standard, 16/190

Manufacture of the candidate standard, 16/190, met the quality control parameters required by WHO. A total of 3814 ampoules were produced. Check-weights measured during filling demonstrated a mean fill weight of 0.4999 g (CV 0.58%, *n* = 12). The mean residual moisture content as determined by coulometric Karl Fischer titration was 1.810% (CV 46.45%, n = 12), mean headspace oxygen was 0.21% (CV 52.37%, n = 12) and the mean dry weight was 0.0030 g (CV 5.78%, *n* = 5).

### Assay validity

In total, data from 42 assay runs from 21 assays methods were submitted to the study. Of these, 19 were different method/platform combinations as shown in Table 2. In accordance with the WHO guidelines for reporting a WHO collaborative study [13], data sets are anonymized as Laboratory methods 1–21 which does not reflect the order of listing in Table 2. The runs of 5 methods (Laboratory methods 8, 10, 11, 17 and 18) were excluded as the slope of the fitted regression line of the log_10_ reported concentration against log_10_ nominal concentration was outside the range [0.91, 1.10]. Two runs (Laboratory method 15, run 2 and method 19, run 2) were excluded as the ratio of the candidate to the coded duplicate of the candidate (A:C) was outside the limits [0.91, 1.10].

**Table 2:** The immunoassay methods and platforms used by to evaluate the proposed reference material for AMH, 16/190

Access2 AMH immunoassay	Beckman-Coulter (performed by two laboratories)
Advia Centaur XP AMH immunoassay	Siemens
Architect i2000sr	Tellgen Corporation
Auto Lumo A2000 plus AMH immunoassay	Autobio Diagnostics Co Ltd
Caris200 AMH immunoassay	Guangzhou Darui Biotechnology Co. Ltd.
CI1000 AMH immunoassay	Beijing Leadman Biochemistry Co.,Ltd.
CIA200 AMH immunoassay	Taizhou Ze Cheng Biotechnology Co
CL2000i AMH immunoassay	Shenzhen Mindray BioMedical Electronics Co Ltd.
Cobas e411 AMH immunoassay	Roche (performed by two laboratories)
Cobas e801 AMH immunoassay	Roche
Dxl AMH immunoassay	Beckman Coulter
Gen II AMH ELISA	Beckman Coulter
iFlash3000	Shenzhen YHLO Biotech Co. Ltd.
Maglumi 4000 plus AMH immunoassay	SNIBE Co. Ltd.
MenoCheck picoAMH ELISA	Ansh Labs
Unicell-S AMH immunochromatography assay	Shenzhen YHLO Biotech Co. Ltd.
Union-CO718 AMH ELISA	Shenzhen YHLO Biotech Co. Ltd.
Vidas 30 AMH fluorescence immunoassay	bioMerieux
Vidas PC AMH fluorescence immunoassay	bioMerieux

The results from each method were assigned a code number which does not reflect the order of listing here

### Value assignment of 16/190 and comparator sample, B

**Fig. 1:**
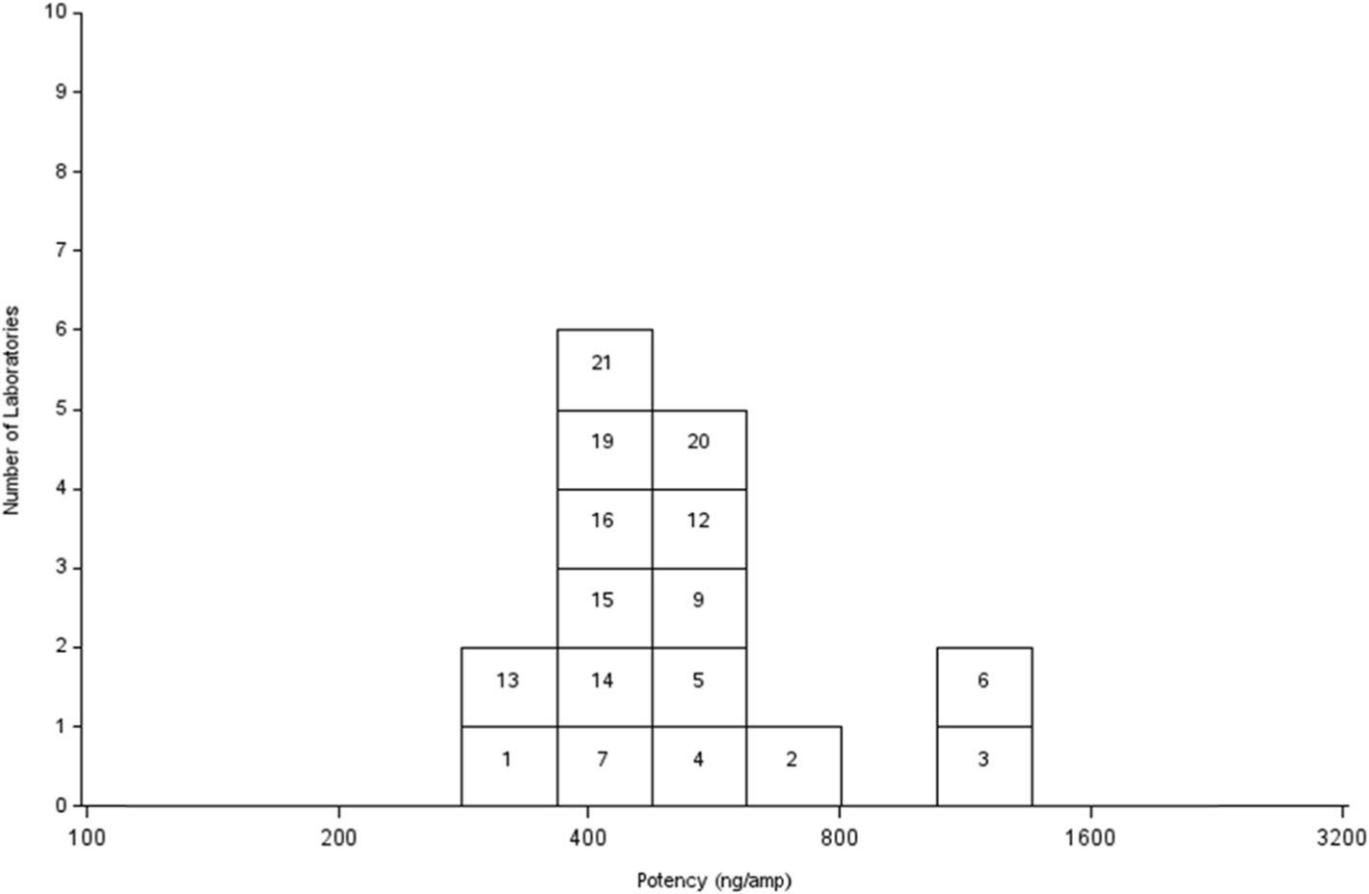
Laboratory geometric mean estimates from valid assays of the AMH content of 16/190

With the exclusions above, 16 methods provided data which met the validity criteria. From these, a geometric mean estimate of the AMH content in 16/190 Sample A and the coded duplicate, Sample C, was determined (Table 3 and Fig. 1). Estimates for the AMH content ranged from 282 ng/ampoule to 1157 ng/ampoule with a geometric mean 511 ng/ampoule (95% CI, 426–612, *n* = 16, GCV 42%) and a robust geometric mean of 489 ng/ampoule. The median estimate was 485 ng/ampoule with an interquartile range of 462–533 ng/ampoule.

**Table 3:** Geometric mean laboratory estimates for the AMH content in ampoules A, B and C as provided by the 16 methods which met the validity criteria

Lab	Sample A	Sample C	GM(A,C)	Sample B	Ratio of B to GM(A,C)
1	337	335	336	150	0.45
2	623	624	624	283	0.45
3	1094	1074	1084	548	0.51
4	523	542	533	293	0.55
5	520	537	528	284	0.54
6	1182	1133	1157	554	0.48
7	484	456	470	252	0.54
8					
9	546	553	549	298	0.54
10					
11					
12	499	506	502	324	0.65
13	277	288	282	141	0.50
14	481	470	475	253	0.53
15	453	471	462	268	0.58
16	461	467	464	203	0.44
17					
18					
19	379	381	380	210	0.55
20	472	498	485	312	0.64
21	410	432	421	228	0.54
Geometric Mean	508	513	511	269	0.53
95% Confidence Limits	417–619	430–612	426–612	221–328	0.50–0.56
GCV	45%	41%	42%	45%	12%
Robust Geometric Mean	487	494	489	265	0.53

Ampoules A and C are coded duplicates of the proposed reference material, 16/190. Ampoule B is a comparator preparation containing approximately half the AMH content

Similarly, geometric mean estimates of the AMH content of the comparator sample B were calculated. These were expressed, for each of the 16 valid laboratory methods, relative to the estimate by that method for 16/190. The overall geometric mean of the potency of B, relative to 16/190, was determined as 0.53 (95% CI, 0.50–0.56, GCV 12%, *n* = 16). Estimates of the AMH content measured in individual runs can be found in report WHO/BS/2019.2363 [18].

### Stability of 16/190

Estimates of the mean relative immunoreactivity of ampoules stored at elevated temperatures were determined from data provided by 5 laboratory methods. As no significant change in relative immunoreactivity was observed in ampoules incubated at elevated temperatures for 21 months and 13 days, 16/190 is likely to be highly stable when stored at − 20 °C.

### Commutability of 16/190

Alongside dilutions of the ampouled preparations, participants were asked to measure, in triplicate, the AMH content of a range of patient samples. These were coded AMHSerum1–3 which were paediatric male serum samples prediluted with a pooled menopausal serum, AMHSerum4, 5, 17 and AMHPlasma21 and 22 from male donors and AMHSerum6–16 and AMHPlasma18–20 from female donors. Of these, 20 samples were included in the analysis of commutability with AMHPlasma18 and 22 being excluded as described above. If the AMH content was outside the range of an assay (laboratory method 1 and 13), participants chose to dilute the sample or to report it as out of range.

The commutability of the candidate standard was assessed at 6 nominal dilution of 0.5, 1, 2, 4, 8 and 16 ng/ml AMH. Where participants measured different dilutions (Laboratory methods 2 and 3, and method 12 in which the specified dilutions were prepared gravimetrically), interpolated values were used. The limits for acceptable bias difference were calculated from the standard deviation of the bias of the serum samples from all laboratories as described above. These were defined as ±0.051, or 0.892 to 1.121 on the untransformed scale, i.e. the bias for a reference standard must be demonstrated to be not less than 89.2% and not more than 112.1% of the bias observed for patient samples. As the mean patient sample bias will be different for each laboratory method, the lower and upper limits that define acceptable commutability will be different for each method. These values are shown in Table 4 which also includes the overall mean bias for the reference material dilutions for each valid method. As this approach requires bias to be constant across the range of AMH concentrations, the slopes of the bias values of the reference material dilutions and patient samples are also shown in Table 4. Individual values and plots of the bias of each patient sample and reference material dilution for each of the 16 valid methods are included in report, WHO/BS/2019.2363 [18].

**Table 4:** Assessment of the commutability of 16/190 with representative patient samples

Laboratory	Mean Patient Sample Bias	Lower Limit	Upper Limit	Mean 16/190 bias	16/190 Slope	Patient Samples Slope
1	−0.029	− 0.078	0.021	− 0.149	1.02	1.04
2	0.137	0.088	0.187	0.143	0.98	1.08
3	0.045	−0.005	0.094	0.348	1.02	0.98
4	−0.014	−0.063	0.036	0.033	1.01	1.04
5	−0.044	−0.094	0.005	0.033	1.00	1.06
6	0.109	0.060	0.159	0.386	1.08	1.07
7	−0.025	− 0.074	0.025	− 0.029	0.97	0.98
9	0.121	0.072	0.171	0.065	0.96	0.95
12	−0.064	− 0.114	− 0.015	0.008	0.99	0.97
13	−0.041	−0.090	0.009	−0.223	1.06	1.06
14	−0.009	− 0.059	0.040	−0.025	1.00	0.97
15	0.023	−0.027	0.072	−0.015	1.02	1.04
16	0.007	−0.042	0.057	−0.002	1.00	0.93
19	−0.078	−0.127	− 0.028	−0.111	1.00	1.03
20	0.042	−0.008	0.091	−0.008	1.00	0.93
21	−0.017	−0.066	0.033	−0.063	1.06	0.97

This shows the mean patient bias for each valid laboratory method, the upper and lower limits of acceptable commutability (±2*s*_*P*_) for each method and the mean bias values for 16/190 obtained by that method. Values underlined indicate where the mean bias is outside the limits of acceptable bias for that method. As this assessment requires the bias to be constant across the range of AMH concentrations measured, the slope of the bias for 16/190 and the patient samples is also shown

As shown in Table 4, the mean overall bias for the reference material dilutions was within the limits of acceptable commutability for laboratory methods 2, 4, 7, 14, 15, 16, 19, 20 and 21. Of these, in three methods (15, 20 and 21), 16/190 was not commutable in 2 or 3 of the test concentrations so could only be considered partially commutable in these methods. This may be due to experimental variation (laboratory method 15 which is the same as method 4), non-constant bias (laboratory method 20) and the freezing of test samples prior to measuring (laboratory method 21 which is the same as method 7).

The candidate standard was not commutable with patient samples when measured by laboratory methods 1, 3, 5, 6, 9, 12 and 13. Of these, laboratory methods 1 and 13 were the same method on different platforms. Laboratory method 5 was the same as method 4 (in which 16/190 was commutable) but on a different platform and although the mean bias for the reference material dilutions was the same by these methods, by method 5, the bias for the patient samples was more negative resulting in the bias for the reference material dilutions being outside the upper limit of acceptable bias. For method 13, the bias values for were non-constant and it may be that this approach is not applicable for this method. An increasingly negative trend in bias was observed for the higher concentrations of 16/190 when measured by method 9.

## Discussion

In response to repeated calls for a WHO IS to calibrate AMH immunoassays, a batch of ampoules, coded 16/190, was prepared by NIBSC and a collaborative study organised to assign a value to the material and to evaluate its suitability. The study, reported here, has provided the first assessment of a stable, lyophilised preparation of recombinant, CHO-derived AMH by multiple immunoassay methods. There are however, limits to the scope of such a study in that each laboratory method is performed only two to three times, rather than multiple times on different days using different instruments, reagent lots and operators as would be required to fully explore within-assay variation. Despite this, it is clear that there is a wide variation in the estimates of the AMH content of 16/190 with estimates ranging from 282 ng/ampoule to 1157 ng/ampoule, resulting in a geometric mean content estimate from valid assays of 511 ng/amp (95% CI: 426–612, *n* = 16, GCV 42%) and a robust geometric mean of 489 ng/amp.

By contrast, measurements of the comparator sample B, containing half the content of AMH, when expressed relative to 16/190, varied with the much lower GCV of 12%. This suggests that the 16 valid laboratory methods exhibited a similar proportionality in their detection of recombinant AMH. As such, possible sources of the observed inter-assay variability in content estimates may be differences in antibody affinity, reaction parameters or assay calibration. Given the unclear traceability of the mass unit of AMH and given that widely discrepant estimates were previously shown to be provided by assays using the same monoclonal antibodies, F2B 12/H and F2B 7/A [11], it is possible that the observed variation is due to differences in assay calibration. It is understandable that, in the absence of a global standard, manufacturers aim to align their methods to currently-available immunoassays to ensure consistency in the measurement of patient samples. We are aware of one manufacturer (Ansh Laboratories) who has pursued SI-traceable value assignment of their calibrators [22]. Other assays have used serum value transfer in which the AMH content of a bank of patient samples, as measured by the current market leading assay, is applied to the signal of the new assay, and therefore to the new calibrators. This approach successfully engineers inter-assay agreement between serum measurements but has the potential to artificially assign a mass value to a set of calibrators. If the calibrator AMH and serum AMH are not recognized by the capture and detection antibodies in an equivalent manner, as may be the case, for example, when bovine AMH calibrators are used, the range of estimates observed in this study is not wholly unexpected.

As expected, this has an impact on the commutability of the candidate reference material. To assess commutability, data from the measurement of the AMH content of 20 patient samples were analysed by an approach similar to that recommended by the IFCC Working Group on Commutability [23]. Of the 16 immunoassay methods which met the validity criteria, 16/190 was within the limits of acceptable bias difference for 6 methods. For a further 3 methods, the mean difference in bias of the nominal dilutions tested, was within acceptable limits for only 3 or 4 of the 6 nominal concentrations assessed. This may be explained by experimental variation, non-constant bias or freeze-thaw of test dilutions. For 7 methods, 16/190 was not commutable. Of these, 2 were the same method on different platforms and 1 was the same method as a commutable method but on a different platform.

As discussed above, the practice of serum value transfer to assign AMH concentration values to assay calibrators of different sources may produce a wide range of responses when presented with a single source of reference standard AMH, in this case human, recombinant AMH. More subtle commutability deviations, particularly where a related platform-assay combination has shown 16/190 to be commutable, may be improved with increased familiarity with the material such as in reconstitution, dilution and examination of the storage conditions of sample dilutions. Lack of commutability in a reference material has often been attributed to the source, matrix and processing that is required to achieve long-term stability in a product that can be readily transported worldwide at reasonable cost to all. It is noted that the concentration of the matrix of 16/190 will be extensively reduced once diluted to the concentrations measured in current assay methods. With regard to the preparation of commutable reference materials, Braga and Panteghini [24] state that ‘the qualification of the measurement procedures about their selectivity should be done independently and in advance’. Thus, it needs to be considered if enough is known about current AMH immunoassays, such as the extent of equivalence of antibody binding to native and calibrator AMH, to consider assessment of commutability appropriate at the current time. Defining the relationship would however, require that validated procedures are available to measure the concentration of the immunoassay calibrators independently of the signal derived from serum value transfer. This will require further technological advances in peptide and glycan identification and quantification.

There is an increasing awareness of the importance of traceability in laboratory medicine such that a measurement result can be related to a reference material through a documented unbroken chain of calibrations [24, 25]. The chain is comprised of a hierarchy of calibrators and measurement procedures, commencing with the Primary Reference material which is defined and measured by a primary reference measurement procedure. It is essential that the reference materials provided at all parts of the traceability chain are commutable. For reference materials calibrated in mass units, the reference measurement procedure must use procedures which are traceable to the SI unit of mass. As mentioned previously, this is a challenge for complex analytes such as glycoprotein hormones which exist in multiple isoforms. For these, international conventional calibrators such as WHO International Standards, have been used successfully to harmonize methods.

The unclear origins of the nanogram of AMH and its subsequent transfer between assays by serum value transfer are clearly far removed from the above metrological ideals for traceability in laboratory medicine. Yet, as discussed previously, there are robust reasons to maintain the accepted units of AMH immunoreactivity. In addition, approval of the Elecsys® AMH assay (Roche) as a companion diagnostic in which the reported AMH concentration is used in an algorithm to calculate the patient dose of Rekovelle®, a follitropin delta product (Ferring Pharmaceuticals) further reinforces the current conventions [26, 27]. Also, this development highlights the challenges of introducing a WHO International Standard, should this result in a requirement for an assay, used as a companion diagnostic, to be recalibrated.

While recognising the issues described above, there is a responsibility to support the current use of AMH immunoassays and to provide a reference preparation which can be used to further investigate assay calibration and the performance of methods.

## Conclusion

This study demonstrated that the majority of AMH immunoassays available for clinical use, recognised recombinant human AMH when reconstituted from the stable, lyophilised form provided in ampoules of the candidate reference material coded 16/190. Estimates of AMH content in terms of individual assay calibration showed considerable variability which may reflect historical calibration exercises and current approaches used to assign values to method calibrators which may be native, recombinant or non-human AMH. However, valid assays exhibited a similar proportionality of response as demonstrated by the low variability of the ratio of content estimates of a comparator sample to the content estimate of 16/190. It was recognised by WHO ECBS that the commutability data of this study would not support the establishment of 16/190 as a WHO International Standard.. To this end, the WHO ECBS approved the establishment of 16/190 as a **WHO Reference Reagent for AMH, human, recombinant**. The status of the material as a WHO Reference reagent, rather than a “full” WHO International Standard, reflects the commutability considerations described above and the resulting intended use as a material to investigate assay performance. Until a physicochemical reference method is available, the AMH content of 16/190 will be defined as 489 ng/ampoule which is the robust geometric mean of the estimates from valid assays. The material is available from NIBSC (nibsc.org).

## Data Availability

The datasets supporting the conclusions of this article can be found at [18].

## Abbreviations

AMH: Anti-Mullerian hormone
CHO: Chinese hamster ovary
ECBS: Expert Committee on Biological Standardization
IFCC: International Federation of Clinical Chemistry
IU: International Unit
NIBSC: National Institute for Biological Standards and Control
SI: Système International
WHO: World Health Organization.
